# Epileptic seizure clustering and accumulation at transition from activity to rest in GAERS rats

**DOI:** 10.3389/fneur.2023.1296421

**Published:** 2024-01-24

**Authors:** Hieu Tran, Reda El Mahzoum, Agnès Bonnot, Ivan Cohen

**Affiliations:** Sorbonne Université, INSERM, CNRS, Neuroscience Paris Seine, Institut de Biologie Paris Seine, Paris, France

**Keywords:** epilepsy, electroencephalogram, electromyogram, electrooculogram, head motion, seizure occurrence, behavior transition, rest

## Abstract

Knowing when seizures occur may help patients and can also provide insight into epileptogenesis mechanisms. We recorded seizures over periods of several days in the Genetic Absence Epileptic Rat from Strasbourg (GAERS) model of absence epilepsy, while we monitored behavioral activity with a combined head accelerometer (ACCEL), neck electromyogram (EMG), and electrooculogram (EOG). The three markers consistently discriminated between states of behavioral activity and rest. Both GAERS and control Wistar rats spent more time in rest (55–66%) than in activity (34–45%), yet GAERS showed prolonged continuous episodes of activity (23 vs. 18 min) and rest (34 vs. 30 min). On average, seizures lasted 13 s and were separated by 3.2 min. Isolated seizures were associated with a decrease in the power of the activity markers from steep for ACCEL to moderate for EMG and weak for EOG, with ACCEL and EMG power changes starting before seizure onset. Seizures tended to occur in bursts, with the probability of seizing significantly increasing around a seizure in a window of ±4 min. Furthermore, the seizure rate was strongly increased for several minutes when transitioning from activity to rest. These results point to mechanisms that control behavioral states as determining factors of seizure occurrence.

## Introduction

In humans, absence epilepsy is characterized by sudden and brief loss of consciousness, correlated with 3–4 Hz bilateral spike–wave discharge (SWD) detectable by scalp electroencephalographic (EEG) recordings, and grouped together to form a seizure. These non-convulsive events affect mostly children and teenagers due to a significant amount of remittance after puberty (1). The attention and learning disorders observed in one-third of young patients as well as the resistance to pharmacological treatment in 30% of cases underline the need to understand its mechanisms and develop new therapeutic approaches (2–5). An approach derived from that used in convulsive epilepsy is to predict seizure occurrence (6, 7) in order to reduce harmful side effects and/or engage in short-term treatment specifically when needed.

Anecdotal descriptions have long suggested an unexplained influence of biorhythms, such as circadian and ultradian, on the modulation of seizure onset, maintenance, and termination. Observation of absence seizures, particularly in children, initially suggested that they occur only during wakefulness, but a growing number of studies have shown that regular SWDs, similar to those of clinical seizures, were also observed during sleep (8, 9). In patients with idiopathic generalized epilepsy (IGE), it was observed that the onset of SWDs would be inhibited by periods of active wakefulness and REM sleep, whereas it would be favored during the transition phase between wakefulness and slow-wave sleep and between slow-wave and REM sleep (10). Thus, the idea emerged that susceptibility to seizure is greater in intermediate states of drowsiness and/or inactivity and lower during conscious physical or mental activity, suggesting that seizure onset could be predicted on the basis of the state of alertness.

Animal models have been pivotal in addressing these questions, revealing specificities both for the duration of states of vigilance and for the genesis of SWDs in relation to these states. In genetic animal models of absence epilepsy, the intermediate stage of sleep, which is a transitional stage occurring before and sometimes after REM sleep and characterized by high voltage spindle activity in frontoparietal areas (11), was found to be almost three times longer in the WAG/Rij rat than in control Wistar rats (12, 13), with an overall shorter sleep cycle (14). In the cat, the efficacy of penicillin to induce SWDs increased during light slow-wave sleep and fell to zero during REM sleep (15). In absence-type IGE rat models, SWDs were predominant during quiet wakefulness and drowsiness and rarely occurred during REM sleep and active wakefulness (14, 16, 17). When WAG/Rij rats were equipped with cortical EEG and nuchal electrodes (18), SWDs were found to occur mainly during light slow-wave sleep and passive arousal and, in contrast, were rare in periods of REM sleep, deep sleep, and active wakefulness. In Smyk’s et al. (19) study using WAG/Rij rats, the authors found that SWDs are most often preceded by active wakefulness and followed by light, slow-wave sleep, regardless of the timing and phase of the circadian cycle. However, in their study, it was impossible to predict SWD onset based on pre-seizure behavioral state, except for a very short time window of 4 s (19).

As SWDs are massively synchronized oscillations occurring selectively in some thalamocortical networks (20), it has been hypothesized that a high degree of desynchronized thalamocortical neuronal activity during periods of active wakefulness and REM sleep would inhibit SWDs (18). However, it is difficult to establish such a direct link between the electrographic manifestations of a condition and the presence or absence of seizures, as SWDs are absent from periods of deep, slow sleep where precisely the degree of synchronization is high.

The hypothesis that seems promising is that of a concordance between the onset of seizure and intermediate levels of alertness, with a partial and/or unstable level of synchronization that can be considered a transition state. SWDs could not only be favored in these periods but also modulate these transitions. There would thus be a functional link between the mechanisms of regulation of wakefulness and seizures. This thesis is widely discussed in Leresche et al. (21) review, which argues that SWDs could be “perverted” physiological sleep clusters, deriving from the 10–14 Hz oscillations called spindles observed mainly during delta light slow-wave sleep and often preceding or following SWDs (21–23). As was already suggested in a study of penicillin-induced generalized epilepsy in cats (24), the excitatory thalamocortical input produced by sleep spindles in the anterior and posterior areas could increase the excitability of cortical neurons and give rise to SWDs in the somatosensory cortex.

For these reasons, in this study, instead of looking for the links between discrete states of vigilance and the appearance of SWDs, we focused on the identification of states of arousal and rest in the GAERS rat. The GAERS rat is a validated genetic model of absence epilepsy (25), as it recapitulates the main markers of this pathology: electrophysiological profile (26), characteristic ethosuximide pharmacological response, behavioral manifestations, and comorbidities (27–29). We identified periods of activity by the level of tonic activity of the neck muscles (EMG), oculomotor muscles (EOG), or by tracking overall head movements using an accelerometer. We asked if there is a preponderance of electrographic seizures during activity, rest, or transitional states. Thus, we addressed the question in terms of how distinct states of alertness may favor seizures.

## Materials and methods

### Animals

For this study, 8 GAERS and 12 Wistar male rats aged 6 weeks to 4 months were used. They were housed under controlled conditions (temperature: 22°C, humidity: 49–52%, day/night cycle: 12 h–12 h, light on at 6 a.m.). Animal care and use conformed to the European Community Council Directive of September 22, 2010 (2010/63/EU) and French law (87/848). Our study was approved by the ethics committee Charles Darwin No. 5 and the French Ministry for Research.

### Electrodes

**Figure 1:**
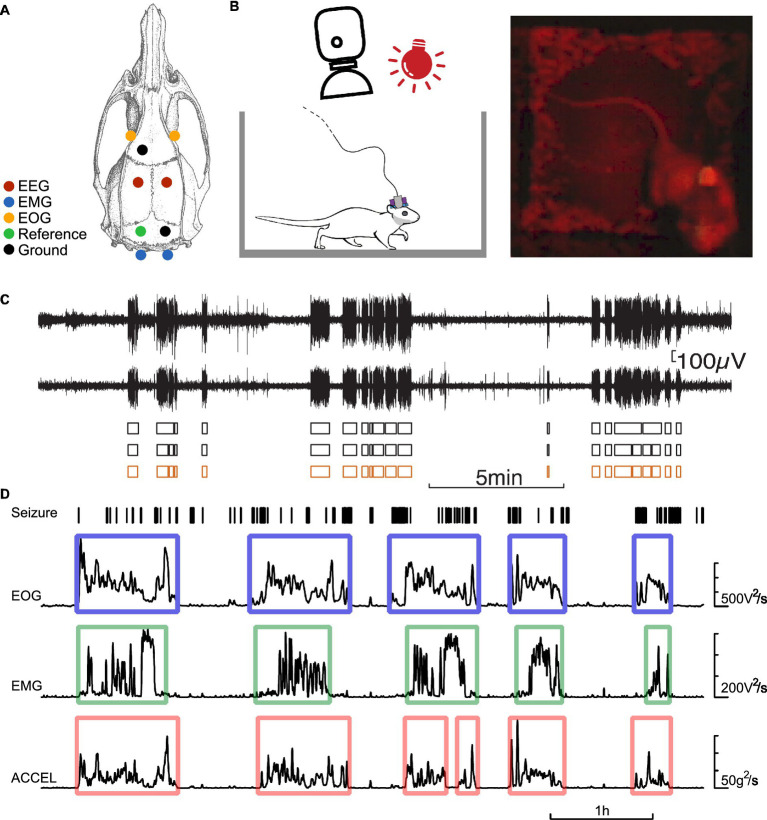
Experimental protocol. **(A)** Dorsal view of the rat skull, illustrating the location of the electrodes. EOG electrodes (orange), EEG bipolar electrodes (red), a reference electrode (green), EMG electrodes (blue), and ground electrodes (black). **(B)** Schematic of the experimental set-up allowing constant video monitoring of the rat equipped with an EEG/EOG/EMG connector and an accelerometer. **(C)** example of bilateral cortical EEG showing seizures (SWD series), their detection on each channel (black boxes), and the consensus seizure state (orange boxes). **(D)** Example of detection of activity and rest periods. From top to bottom: seizures detected from EEG, power of EOG, EMG signals pre-filtered high-pass at 100 Hz, power of accelerometer signal pre-filtered bandpass at 0.3–3 Hz. Power signals are smoothed over 15 s. The contours indicate the periods of activity detected above a threshold for the power of each signal.

The five-channel EEG electrodes were made of 50 μm diameter tungsten wire. For each electrode, five wires were soldered to five miniature connectors. Their tips had a difference in depth of 0.5 mm. For the EOG (bipolar, 50 μm diameter tungsten wires) and EMG (bipolar, 25 μm diameter tungsten wires) electrodes, the wires were inserted into the periorbital muscle and the neck muscle, respectively. An EEG electrode implanted on the surface of the cerebellum served as a reference electrode. Ground electrode screws were attached above the olfactory bulb and the cerebellum. The locations of the electrodes are shown in Figure 1.

### Surgery

Anesthesia was induced by inhalation of isoflurane for 20 min at 2% and then maintained by intramuscular injection of a ketamine/xylazine mixture (80/10 mg/kg). The animal was placed in a stereotaxic frame on a heating mat (Bioseb, France) to maintain a temperature of 36°C. Under general anesthesia, an incision was made along the sagittal axis of the head. A pair of five-channel electrodes was placed bilaterally at the level of the primary somatosensory cortex via two small holes drilled in the cranial bone at stereotaxic coordinates taken relative to the Bregma: −1.3 mm in the anteroposterior (AP) axis; 5.0 mm in the mediolateral (ML) axis; and − 3.0 mm in the dorsoventral (DV) axis (30). Electrodes for EOG, EMG, reference, and ground were placed in corresponding muscles. The electrode assemblies were secured to the skull with dental cement. Analgesia was provided in the post-surgical period (buprenorphine 0.1 mg/kg every 12 h for 48 h). Three nuts were sealed in the cement to allow attachment of the accelerometer. Surgery recovery rest was at least 3 days.

### Recording

Simultaneous recordings (Figure 1) of EEG, EMG, EOG, and head acceleration (ACCEL) in GAERS and Wistar rats were obtained for 116 ± 43 (n = 8 rats) and 107 ± 44 (n = 12 rats) h, respectively (AVG ± SD). A brief (15–30 min) isoflurane induction is allowed to attach the connector on the head of the animal to the acquisition system, composed of a high input impedance amplifier (high-pass filter at 1 Hz, gain of 1,000) and a digitizer (Xcell, Dipsi, Cancale, France).

### Detection of activity and rest periods

The activity and rest periods were determined according to three independent criteria based on EOG, EMG, and ACCEL signals (Figure 1). The EOG and EMG signals were high-pass filtered at 100 Hz, and then the instantaneous power (square) was computed and smoothed by convoluting a Gaussian function of time constant σ of 15 s. Accelerometer signal on each of the three axes was bandpass filtered between 0.3 and 3 Hz, and then the instantaneous power (square) was computed and smoothed by convoluting a Gaussian function of time constant σ of 20 s. The ACCEL signal was then computed by summing the three axes. EOG, EMG, and ACCEL signals showed rapid transitions between stable periods near zero (rest) and periods of large fluctuations (activity). The periods of activity were detected by a threshold above the noise level on the power signals set to correspond to transitions between near-zero power and large fluctuations. On EMG and EOG signals, a minimum duration of 3 min was required to validate an activity period, and a tolerance of 5 min below the threshold was allowed to consider that the animal was still in the same activity cycle. This avoided splitting activity periods due to short breaks in behavior. Rest periods were taken between two consecutive activity periods. On the ACCEL signal, a minimum duration of 2 min was required to validate an activity period, and the tolerance was 5 min below the threshold.

### Detection of absence seizures

Absence seizures were detected by means of a succession of processing of the EEG signal (Figure 1): bandpass filtering, detection of SWDs and series of SWDs on individual channels, and the presence of concomitant series of SWDs on at least half of the channels as a sign of seizure. First, the raw signals were filtered in the 5–50 Hz bandwidth to preserve the SWDs. SWDs were detected on the basis of their amplitude, typically in the range of 150–1,500 μV. A series of SWDs were detected on each channel based on the following criteria: a maximum interval between SWDs of 1 s, an average interval between SWDs of at least 0.4 s, and a duration of the series of SWDs of at least 2 s. Seizures were identified when at least half of the channels showed a series of SWDs. However, if this condition was not met for a short period of <2 s, then the algorithm ignored the gap. In this way, seizures were not split into pieces in case of brief fluctuations in the SWD pattern. The minimum duration for seizure was set at 3 s.

Detected seizures were used to compute the seizing time fraction in activity or rest episodes as the ratio of time spent in seizure over the whole duration of the episode. A seizing state function was defined as having a value of 1 during a seizure and 0 in the interictal periods.

### Statistical analysis

Comparisons were based on the Student’s *t*-test, considering means and standard deviations (unpaired two-sided *t*-test). Correlations were tested using the Fisher *z*-test on the Spearman correlation coefficient since the underlying distributions of durations were not Gaussian. Peri-event graphs were computed by aligning all occurrences of the events, such as activity–rest transition, seizure initiation, or termination. For each time delay around the reference event, we computed the mean and SEM of the observed signal. Signal processing and statistical analyzes were performed with LabVIEW (National Instruments).

The raw data supporting the conclusions of this article will be made available by the authors without undue reservation.

## Results

The activity of GAERS rats was continuously monitored for several days. Our protocol recorded 16,481 seizures (n = 8 rats, 939 h total recording time), which were consistent across animals. The activity level of these animals was simultaneously recorded and compared to control non-epileptic Wistar rats following the same recording procedure (*n* = 12 rats, total 1,288 h).

### Activity and rest in GAERS and control animals

We wished to test whether activity and rest could show a temporal relationship to seizures. As detailed in the Methods section, we considered three criteria to estimate the activity/rest state of the animal: motion of the head sensed by an accelerometer, tonus of the neck, and eye motion estimated from electromyograms of corresponding muscles. For each criterion, we quantified the power intensity of the signal at each point in time. During activity, these signals show a sharp rise above the baseline that is observed when the animal is idle (Figure 1). We could determine the time windows of activity and rest by applying a thresholding procedure to these activity markers.

These three criteria measure some aspects of activity and rest, which should overlap most of the time but not always. For example, an animal may remain motionless (ACCEL at rest state) for some time while otherwise alert, showing active EMG when neck muscles maintain head position. Yet, recordings showed that the criteria matched most of the time. We quantified this observation over all our recordings by counting the fraction of time one criterium is in active or rest state versus another criterium (Table 1). Most often (81.5–88.6% of the time), any two criteria matched, being simultaneously in the active or rest state.

**Table 1:** ACCEL/EMG/EOG match in fraction of time (AVG ± SD).

GAERS, % time, *n* = 8 recordings			Match	Wistar, % time, *n* = 12 recordings			Match
	EMG on	EMG off			EMG on	EMG off	
ACCEL on	31.8 ± 7.9	1.6 ± 1.7	88.6 ± 5.6	ACCEL on	30.5 ± 3.9	2.8 ± 1.9	86.4 ± 4.8
ACCEL off	9.9 ± 6.5	56.8 ± 9.3		ACCEL off	10.8 ± 5.6	55.9 ± 6.7	
ACCEL on	24.9 ± 7.6	8.4 ± 6.6	83.7 ± 6.9	ACCEL on	28.5 ± 4.0	4.7 ± 3.1	86.1 ± 4.6
ACCEL off	7.9 ± 4.7	58.8 ± 9.5		ACCEL off	9.2 ± 5.0	57.5 ± 3.7	
EMG on	28.0 ± 8.3	13.7 ± 8.7	81.5 ± 8.7	EMG on	31.8 ± 5.4	9.5 ± 5.5	84.6 ± 5.9
EMG off	4.8 ± 4.8	53.5 ± 10.7		EMG off	5.9 ± 3.8	52.8 ± 7.0	

**Figure 2:**
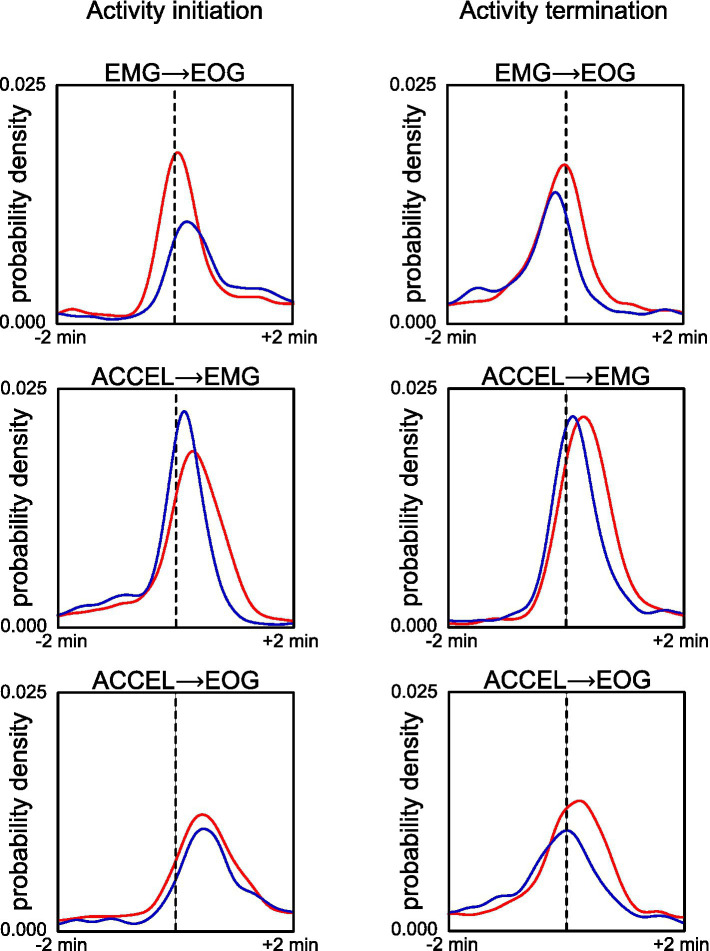
Comparison of fine timing of activity initiation (left panels) and termination (right panels) across criteria for GAERS (blue) and Wistar (red). Reference time (dashed line) is defined as the transition according to EMG criteria (upper plots) or ACCEL criteria (middle and lower plots). Density plot curves are computed for transition according to EOG (upper and lower plots) or EMG (middle plot).

We compared the initiation and termination of activity according to our criteria (Figure 2; Table 2). Pairwise comparison between any two criteria showed marked peak probability at a latency of <30 s. In Wistar, activity beginning and end have a similar sequence: ACCEL is the first, followed after 13–27 s by EMG and EOG, which are in a short succession (−2 to 3 s). In GAERS, activity starts with a 29 s sequence of almost equally spaced ACCEL, EMG, and EOG. Activity ends in a shorter sequence (11 s), in the order EOG, ACCEL, and EMG.

**Table 2:** Delay of the peak probability density function for activity initiation and termination.

		Start delay (s)			End delay (s)		
		Half height before	pdf peak	Half height after	Half height before	pdf peak	Half height after
Wistar	EMG → EOG	−18	**3**	27	−32	−**2**	22
	ACCEL→EMG	−9	**17**	52	−11	**18**	49
	ACCEL→EOG	−5	**27**	68	−24	**13**	49
GAERS	EMG → EOG	−10	**13**	44	−36	−**11**	10
	ACCEL→EMG	−12	**8**	31	−17	**7**	32
	ACCEL→EOG	0	**29**	60	−40	**1**	34

For the curves of Figure 2, we measured the delay in seconds for the half-height before the peak, for the peak, and for the half-height after the peak.

**Figure 3:**
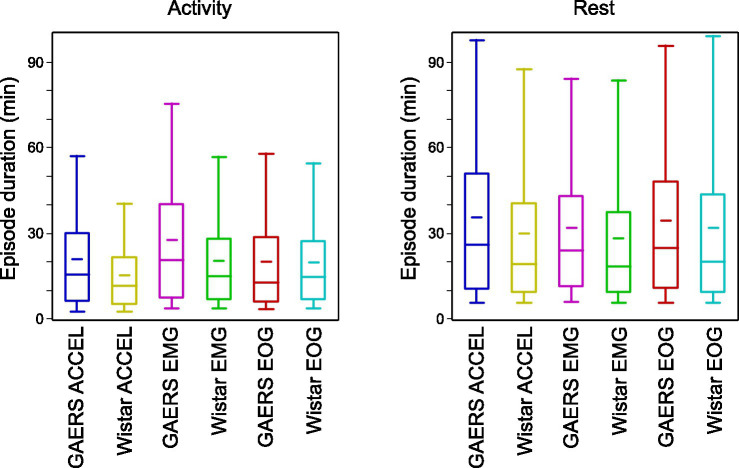
Duration of activity and rest periods. Box plots of activity and rest duration, in GAERS and Wistar, for each of the three criteria. Box plots show 5, 25, 50, 75, and 95 percentiles. The narrow bar represents the mean.

We measured the time an animal spends continuously in an active or resting state and the overall fraction of time in the two states (Table 3). Numbers were consistent across criteria, with both GAERS and controls spending a larger fraction of time in rest (54.7–66.5%) than in activity (33.5–45.3%), with longer continuous episodes of rest (28.3–35.6 min) than activity (15.3–27.6 min). We compared the duration of continuous episodes between GAERS and control (Figure 3). Unlike the overall fraction of time spent at rest and in activity, which did not differ between the two groups, the duration of continuous episodes was significantly longer for the GAERS group compared to the control group (one-sided Student’s *t*-test, ACCEL activity *t* = 8.242, *p* < 0.001; ACCEL rest *t* = 4.539, *p* < 0.001; EMG activity *t* = 7.742, *p* < 0.001; EMG rest *t* = 3.138, *p* < 0.001; EOG rest *t* = 1.982, *p* < 0.025), except for EOG activity. Thus, for most criteria, the GAERS group showed less fragmented patterns of activity and rest while keeping the same overall fraction of time in active and rest states. When we combine the three criteria for continuous episodes, we obtain a simplified overview of 22.8 vs. 18.5 min for activity and 34.0 vs. 30.1 min for rest, for GAERS vs. Wistar, respectively.

**Table 3:** Time in activity and rest.

		GAERS activity	GAERS rest	Wistar activity	Wistar rest
ACCEL	min	20.9 ± 18.9, *n* = 988	35.6 ± 31.3, *n* = 996	15.3 ± 13.5, *n* = 1,687	30.1 ± 28.4, *n* = 1,699
	%	36.3 ± 4.6, n = 8	63.6 ± 4.6, *n* = 8	33.5 ± 3.5, *n* = 12	66.5 ± 3.5, *n* = 12
EMG	min	27.6 ± 25.4, *n* = 938	32.0 ± 29.4, *n* = 946	20.3 ± 17.6, *n* = 1,566	28.3 ± 27.4, *n* = 1,578
	%	45.3 ± 7.0, *n* = 8	54.7 ± 7.0, *n* = 8	41.6 ± 7.6, *n* = 12	58.4 ± 7.6, *n* = 12
EOG	min	20.0 ± 18.8, *n* = 1,020	34.5 ± 32.0, *n* = 1,028	19.8 ± 17.1, *n* = 1,476	32.0 ± 31.5, *n* = 1,488
	%	35.9 ± 8.9, *n* = 8	64.1 ± 8.9, *n* = 8	38.0 ± 5.5, *n* = 12	62.0 ± 5.5, *n* = 12

Duration of episodes of activity and rest in minutes, AVG ± SD. Fraction of time (%) across a recording.

Since the duration of an episode could be influenced by the previous one, as in sleep inertia (31), the relationship between the duration of a period of activity (respectively rest) and the preceding period of rest (respectively activity) was explored in Table 4. In both Wistar and GAERS strains, correlation coefficients (Pearson’s R) range from −0.045 to 0.101, meaning that the duration of one period has little effect on the duration of the next, explaining less than 10% of its variance. Despite their low values, we found correlations to be significantly non-null, mostly for GAERS (*p* <0.05), but not for Wistar (*z*-test on Spearman’s *ρ*).

**Table 4:** Correlation between the duration of consecutive rest and activity periods.

	ACCEL				EMG				EOG			
	R	*n*	*ρ*	*p*	R	*n*	*ρ*	*p*	R	*n*	*ρ*	*p*
Wistar rest vs. previous activity	0.013	1,687	0.040	0.098	−0.045	1,566	−0.048	0.056	−0.008	1,476	0.003	0.941
Wistar activity vs. previous rest	0.028	1,699	0.021	0.394	−0.004	1,578	−0.020	0.425	0.033	1,488	0.030	0.241
GAERS rest vs. previous activity	0.080	988	0.081	0.011	0.078	938	0.121	<0.001	0.078	1,020	0.132	<0.001
GAERS activity vs. previous rest	0.072	996	0.121	<0.001	0.101	946	0.179	<0.001	0.065	1,028	0.121	<0.001

Pearson’s coefficient, *n*, Spearman’s *ρ*, and *p*-value for *z*-test *ρ* ≠ 0.

### Seizures in activity and rest

**Figure 4:**
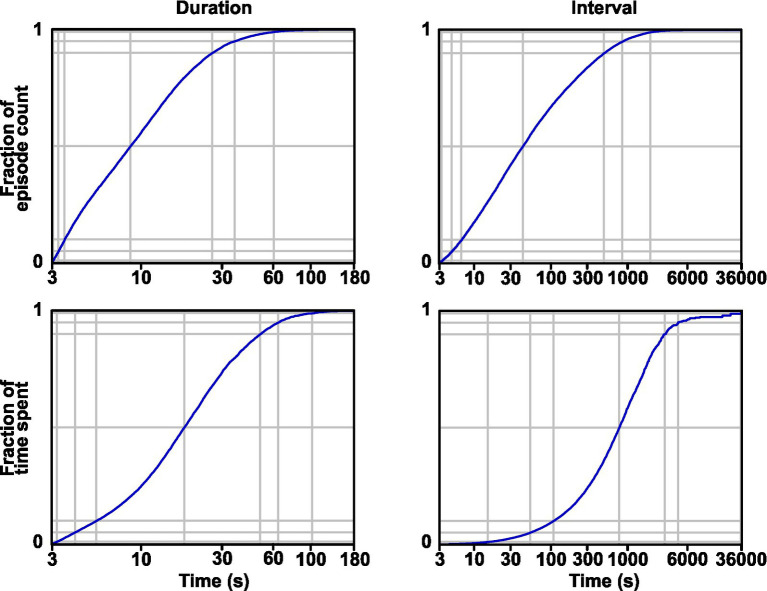
Distribution of seizure duration and interval. Cumulative distributions are shown in terms of the count of events **(A)** and time spent **(B)**. Soft gray lines show the 1, 5, 25, 50, 75, 95, and 99 percentiles from bottom to top.

Given that GAERS and control rats exhibit distinct activity and rest patterns, we wondered whether this particularity of the GAERS rat could be linked to seizure generation. Thus, we explored the occurrence of seizure in relation to the activity–rest cycle. First, taking into account both seizures occurring during activity and rest, we computed the distribution of seizure durations and the distribution of intervals between consecutive seizures (Figure 4, upper part). As expected, these two variables showed a steady decline for larger times (Figure 4; duration: 12.7 ± 12.0 s, AVG ± SD, n = 16,481 seizure; interval: 3.2 ± 9.5 min, AVG ± SD, n = 16,473). More specifically, the median seizure duration was 9 s, 95% of seizures were shorter than 36 s, and 99% were shorter than 60 s. We also computed these two distributions relative to time spent rather than a count of events (Figure 4, bottom part). Thus, half of the seizure time was spent in seizures shorter than 18 s, 95% of time in seizures shorter than 65 s, and 99% of time in seizures shorter than 102 s. The median value for seizure interval was 39 s, 95% were shorter than 14.8 min, and 99% were shorter than 36 min. Half of the non-seizing time was spent in intervals shorter than 13.1 min. Thus, while the intervals between seizures are typically several minutes, which is in the range of the duration of activity and rest episodes, seizures are, on the contrary, typically shorter than a minute, which is much shorter than continuous episodes of activity or rest.

How do seizures compare between activity and rest periods? In GAERS rats, we compared the duration of seizures and the overall fraction of time spent seizing in activity and rest (Table 5). Seizure length is slightly yet significantly shorter during activity compared to rest using ACCEL and EOG criteria (11.8 ± 10.4 s vs. 13.4 ± 13.2 s, *p* < 0.001; 10.4 ± 9.8 s vs. 14.2 ± 13.0 s, Student *p* < 0.001), but not EMG. The fraction of time spent seizing is also slightly but significantly shorter during activity compared to rest using ACCEL and EOG (7.5 ± 8.2% vs. 8.4 ± 12.4%, *p* < 0.03; 5.4 ± 6.8% vs. 8.2 ± 10.5%, Student *p* < 0.001). However, the seizing time fraction was longer in activity compared to rest using the EMG criteria (8.2 ± 9.2% vs. 6.0 ± 10.3%, Student *p* < 0.001). This difference between criteria could be caused by the occasional criteria mismatch (Table 1) or by the delays in transition times (Figure 2).

**Table 5:** Time spent in seizure during activity and rest.

	Activity	Test	Rest
ACCEL			
Duration (s)	1,248 ± 1,129		2,124 ± 1879
Seizing time (s)	88 ± 120		120 ± 156
Seizing fraction (%)	7.5 ± 8.2	*p* < 0.03	8.4 ± 12.4
*n* period	1,004		1,009
Seizure duration (s)	11.8 ± 10.4	*p* < 0.001	13.4 ± 13.2
*n* seizure	7,409		9,072
EMG			
Duration (s)	1,646 ± 1,517		1904 ± 1764
Seizing time (s)	137 ± 196		82 ± 124
Seizing fraction (%)	8.2 ± 9.2	*p* < 0.001	6.0 ± 10.3
*n* period	954		959
Seizure duration (s)	12.7 ± 11.8	NS	12.6 ± 12.4
*n* seizure	10,532		5,949
EOG			
Duration (s)	1,197 ± 1,126		2067 ± 1928
Seizing time (s)	67 ± 94		133 ± 191
Seizing fraction (%)	5.4 ± 6.8	*p* < 0.001	8.2 ± 10.5
*n* period	1,036		1,043
Seizure duration (s)	10.4 ± 9.8	*p* < 0.001	14.2 ± 13.0
*n* seizure	6,665		9,816

Duration of activity and resting periods using the 3 criteria ACCEL, EMG, EOG. Time in seizure in activity and rest. Ratio of time spent seizing in activity and rest periods. Distribution of fraction of time seizing. One-sided Student’s *t*-test.

We then wondered whether the seizing time fraction in one phase (activity or rest) was significantly influenced by the seizing time fraction in the previous phase (respectively, rest or activity). To do this, we compared the seizing time fraction of consecutive periods in the cycle of activity and rest (Table 6). All six correlation coefficients were significantly non-null (*z*-test on Spearman’s *ρ*). The correlation coefficients (R) were all positive and similar for the three criteria between the seizing fractions in rest and in the preceding activity, and in the range of 0.233–0.360. The correlation coefficients were smaller and in the range of 0.073–0.167 between the seizing fraction in activity and in the preceding rest. We compared the pairs of correlation coefficients (rest vs. activity and activity vs. rest) for each of the three criteria and found the difference to be significant for ACCEL and EMG but not for EOG (*z*-test on Spearman’s ρ: ACCEL: *p* < 0.001; EMG: *p* < 0.001; EOG: *p* = 0.034). Thus, at least when ACCEL and EMG are considered, the fraction of time spent seizing in activity explained more variance of the fraction of time seizing in the following rest period than the other way around.

**Table 6:** Seizing fraction correlation between consecutive activity and rest periods.

	ACCEL				EMG				EOG			
	R	*n*	*ρ*	*p*	R	*n*	*ρ*	*p*	R	*n*	*ρ*	*p*
Rest vs. previous activity	0.287	998	0.386	<0.001	0.360	947	0.541	<0.001	0.233	1,029	0.399	<0.001
Activity vs. previous rest	0.073	1,002	0.235	<0.001	0.166	951	0.337	<0.001	0.167	1,035	0.318	<0.001

Pearson’s coefficient, *n*, Spearman’s *ρ*, and *p*-vaue for *z*-test *ρ* ≠ 0.

### Relation between seizure and activity markers

**Figure 5:**
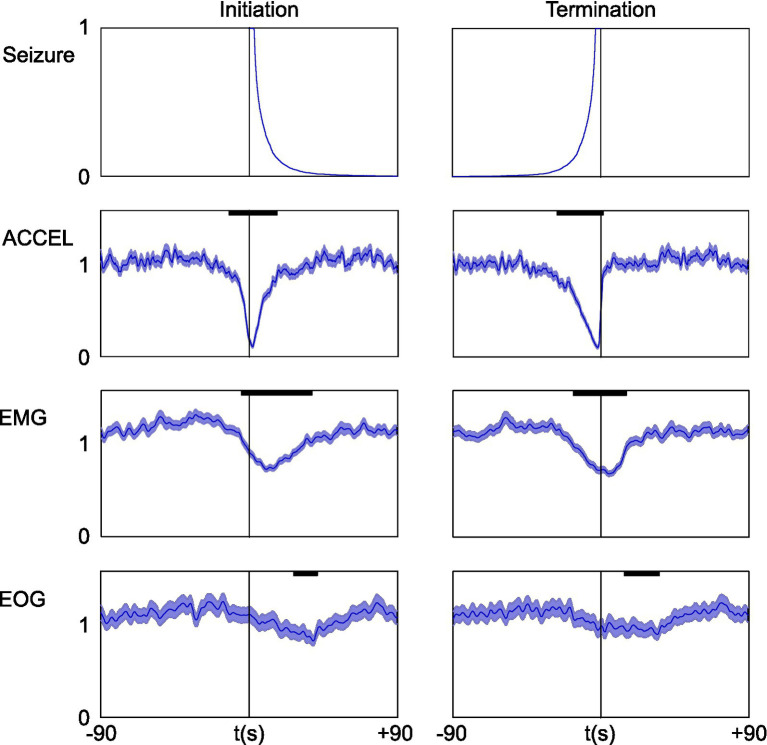
Short-term effect of seizure on activity and power signals. Data were aligned on seizure initiation (left) or termination (right). Upper plot shows the average of the seizing function. For clarity of interpretation, only time windows with a single seizure were used. Below: ACCEL/EMG/EOG power average ± SEM. For better time accuracy, power was smoothed with a shorter time constant of Tau = 1 s. Significant effects (bold black lines) last tens of seconds (*p* < 0.01).

Since electrographic absence seizures in GAERS are known to be associated with freezing behavior, we quantified the effect of seizure on our activity markers (Figure 5). We aligned the data on seizure, either initiation or termination, to compute the average time course of the power signal on ACCEL, EMG, and EOG over a time window of ±90 s. Only time windows with a single seizure were used so that statistics were not biased by seizures occurring at short interval. All three activity markers showed a similar profile between the initiation and termination of seizure, with a decrease in their power. The effect was strong for ACCEL, moderate for EMG, and weak for EOG (respectively peaking down to 0.12, 0.62, and 0.76 of baseline during initiation and down to 0.12, 0.61, and 0.83 of baseline at termination). Delays relative to the onset of seizure were also distinct, with respective significance (*p* < 0.01) in the range − 15 to +19 s, −5 to +41 s, +17 to +48 s for initiation, and − 30 to +1 s, −20 to +26 s, −7 to +40 s for termination, for ACCEL, EMG, and EOG, respectively. Interestingly, ACCEL and EMG power showed a significant decrease before seizure onset, suggesting that seizure is not causal to the freezing behavior. The EOG slight decrease in activity displays a different timing, occurring rather outside of the seizing period.

### Seizure occurrence depends on recent seizure state

**Figure 6:**
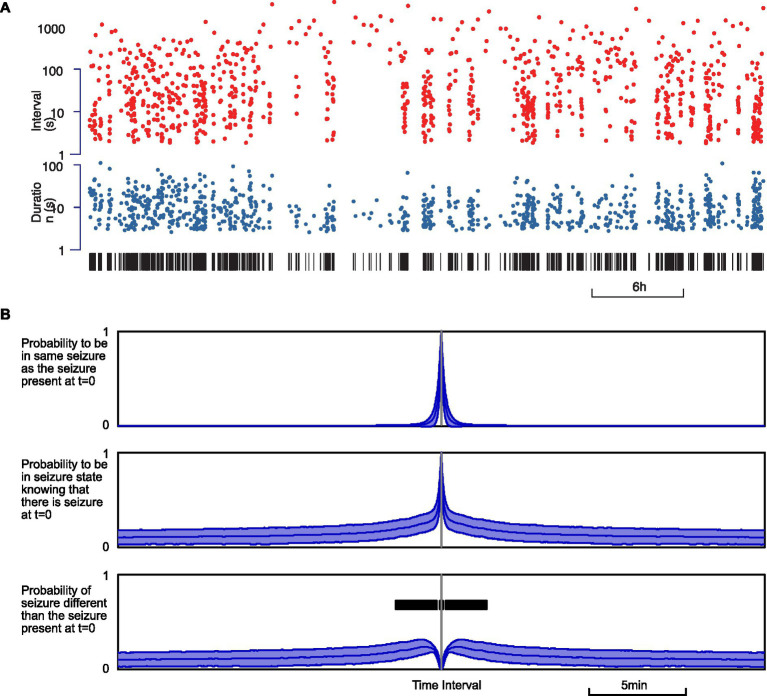
Seizure occurrence process is not time-independent. **(A)** Example of seizure intervals (top, red) and durations (below, blue) over a 30-h recording. Seizures are indicated with vertical black bars below. **(B)** Estimated conditional probability of a seizure state when the animal is in seizure at *t* = 0. From top to bottom: probability of the same seizure; probability of any seizure; probability of a seizure different from the seizure at *t* = 0. The thickness of the curves represents the standard error. The bold black bar indicates the time window when the increase is significant relative to baseline (*p* < 0.01).

One of the parameters likely to influence the next occurrence of an epileptic seizure, in any phase, is the number of recent past seizures. Indeed, it has been known for decades (32) that seizures can show a palette of temporal distribution, from time-independent process (Poisson) to non-Poisson process exhibiting seizure clusters. In our recordings, seizures seemed to occur as a non-time-independent process (Figure 6). This was most visible when seizures were marked as a pair of dots for duration and interval, which showed patterns previously coined as “palisade” in seizures observed in epileptic patients (33). These patterns correspond to clusters of seizures of varying length coming in short succession, interspersed with variable and sometimes prolonged interictal periods (Figure 6A).

In order to quantify seizure clustering, we measured the probability of being in a state of seizure at any time *t* before or after being in a seizure state at a reference time *t* = 0. First, we estimated the probability of being in a state of seizure originating from the same seizure that was present at *t* = 0. Then we estimated the probability of seizing at time *t* due to the seizure at *t* = 0 or any other seizure. By subtracting the two, we obtained the probability of seizing at time *t* from a seizure distinct from the one at *t* = 0. If seizures were to occur independently, this probability should be flat, except for intervals on the order of magnitude of the duration of a seizure (12.7 ± 12.0 s). Indeed, the simple fact that seizures have a non-null duration prevents the short-term occurrence of a distinct seizure. This time window of reduced occurrence of distinct seizure corresponds to the upward peak observed in the upper probability curve and the downward peak at zero in the lower probability curve (Figure 6B). We observed that the empiric probability of a distinct seizure flattens as expected for long intervals, above 5–10 min, indicative of time independence only for large intervals. Yet we found significant changes (*p* < 0.01) in the interval − 4.25 to +4.25 min, which suggests that seizures tend to cluster in increased seizing bouts of more than 4 min.

### Seizure occurrence at transition from active to rest state

**Figure 7:**
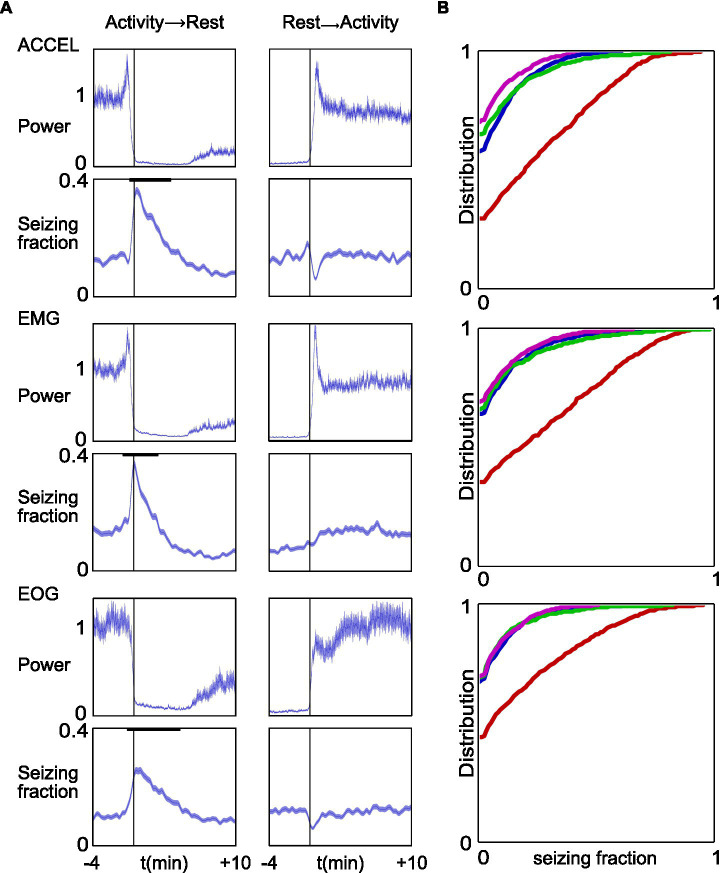
Increased fraction of time spent seizing around transition time from activity to rest. **(A)** Top to bottom panels represent the same computation for ACCEL, EMG, and EOG, respectively. Data were aligned on the reference transition time (left, activity to rest; right, rest to activity). In each panel, upper plots show the mean and SEM of the corresponding power signal, normalized as 1 for baseline values. Lower plots show the mean ± SEM of the seizing function. Bold black lines indicate windows of significant variation at *p* < 0.01. **(B)** Cumulative distribution of seizing fraction in a 1-min interval centered around transition from activity to rest (red), transition from rest to activity (blue), activity midpoint (green), and activity maximum (pink).

Finally, we wondered whether there was a temporal relationship between the occurrence of seizure and the activity/rest cycle. We aligned data on the transition time from activity to rest and from rest to activity, determined according to one or other of the three criteria. We could thus estimate the average, over all our recordings, of the seizure state function, which values 1 during a seizure and 0 in the interictal period, over a time window of 4 min before and 10 min after each transition. As a comparison, we also computed the average power in the continuous signals used to detect activity periods (ACCEL, EMG, and EOG) (Figure 7).

We found a strong and prolonged increase in seizure density at the transition from activity to rest (ACCEL: from −20 s to +4.4 min, *p* < 0.01 *n* = 998, peaking at 0.343 from a baseline at 0.077; EMG: from −29 s to +2.4 min, *p* < 0.01 *n* = 949, peaking at 0.363 from a baseline at 0.088; EOG: from −34 s to +6.0 min, *p* < 0.01 *n* = 1,030, peaking at 0.230 from a baseline at 0.058). Thus, seizure fraction increased by 4.5×, 4.1×, and 4.0× for ACCEL, EMG, and EOG criteria, respectively. A minor dip in seizing time fraction was observed before transition time only for ACCEL (from −39 s to −27 s, *p* < 0.01 *n* = 998, trough at 0.023 from a baseline at 0.077).

At the transition from rest to activity, we found a small dip in seizure fraction average according to ACCEL and EOG criteria (ACCEL: from 14 s to 56 s, *p* < 0.01 *n* = 998, trough at 0.044 from a baseline at 0.077, a 43% decrease; EOG: from −16 s to −71 s, *p* < 0.01 *n* = 1,033, trough at 0.026 from a baseline at 0.079, a 67% decrease). A minor increase observed for the EMG criteria was not significant. A minor increase was observed just prior to the transition for the ACCEL criteria (from −32 s to −4 s, *p* < 0.01 *n* = 1,033, peaking at 0.125 from a baseline at 0.077).

These results suggest that seizures tend to accumulate at the transition from activity to rest and are slightly less frequent at the transition from rest to activity. This observation was further supported when we computed the distribution of seizing fraction in a time window of 1 min centered on activity/rest transitions and on distant time points used as comparison (Figure 7B). The transition from activity to rest stands out as having about half the proportion of complete absence of seizure (null fraction) and an enhanced contribution of larger seizing fractions.

## Discussion

In this study, we compared markers of activity and rest between absence epilepsy and control rats, and we analyzed the temporal pattern of seizure in relation to activity and rest. The three markers that we used (ACCEL, EMG, and EOG) were consistent and showed transition times between activity and rest that matched within half a minute. Epileptic and control animals exhibited the same proportion of activity and rest. The duration of continuous episodes of activity and rest were on the order of tens of minutes and were longer in the epileptic animal than in control. Seizures were typically much shorter than periods of activity or rest, lasting less than a minute. On average, a single seizure occurred concomitantly with a partial and transient decrease in the power of activity marker signals. Spontaneously occurring seizures tended to come in clusters, corresponding to a time window of around 4 min. Seizing time fraction was found to be increased by more than 4-fold at the transition from activity to rest.

### Relevant criteria for determining behavioral state

In this study, we have chosen to compare seizure occurrence with the global level of activity, rather than circadian rhythm or vigilance states (19, 34). Despite extensive efforts, these authors could not predict seizure occurrence based on the pre-seizure state. Similarly, no clear link between circadian rhythms and seizure onset could be identified. Our rationale was that lab rodents have only partial coupling to circadian rhythms and that extensive distinction between the different resting states (quiet wakefulness and sleep stages) could blur the overall picture. Furthermore, in humans, absence epilepsy triggers gaps in consciousness, including loss of environmental awareness and interruptions in behavior, and we wanted to use this solid behavioral marker as a starting point for our analysis. For this reason, we considered behavioral activity parameters *en bloc* to detect periods of activity, and all remaining periods were considered as rest.

Our markers for activity were overall consistent (Table 1), with a good match for phase start and end (Figure 2; Table 2). Differences between any two criteria for activity may correspond to specific behaviors, for example, alert immobile posture (ACCEL low with EMG and EOG high), relaxed head (EMG low and EOG high), or relaxed gaze (EOG low and EMG high) during wakefulness, postural shift motion during sleep (ACCEL high and EMG low), or eye movement in paradoxical sleep (EOG high with EMG and ACCEL low). Our criteria were therefore precise and could reveal subtle behavioral differences. Yet in this study, we did not try to correlate them systematically with behavior, and we used them mainly so that their common activity or inactivity allowed us to represent activity and rest phases in a binary way.

### Candidate mechanisms that could explain the seizure pattern

The pattern of occurrence of seizures constrains the candidate mechanisms of their generation and sheds light on the physiological processes that epilepsy may build upon.

The first major element that we discovered in this model of absence seizure is the clustering pattern on a time scale of around 4 min, which suggests that a specific mechanism generating bursts of seizures modulates ictogenesis. This duration is much longer than the average seizure, implying that seizures are not merely independent events, each with their sole generation process, although there are mechanisms at this time scale (34, 35). Rather, seizures appear to ride upon a more massive underlying mechanism of “epileptic storm,” carrying a series of seizures and short lulls. This underlying mechanism responsible for seizure burst generation (SBG) lays at an intermediate level between those of ictogenesis, on the scale of a few tens of seconds, and epileptogenesis, on the scale of days to months. In addition to the distinct time scales, the observation that many seizures occur without eliciting rest and that lone seizure have only a limited effect on some activity markers (Figure 5), while the transition from activity to rest is associated with a strong and prolonged increase in seizing fraction in line with seizure bursts (Figure 7), further supports the existence of a specific mechanism for SBG.

Our second major observation, that seizing increases strongly at transitions from activity to rest, suggests that SBG is linked to circuits of the activity/rest cycle, a component of metabolic homeostasis. Studies on the WAG/Rij model have reported that SWDs tend to occur in transitional states, around sleep onset and awakenings (18). Our study confirms the importance of transitional states in seizure generation, suggesting that cerebral state switching networks, comprising thalamus, striatum, or brainstem nuclei, could be dysfunctional or not timely activated, as hypohesized in WAG/Rij (36). Our data further show that the activity–rest transition is not symmetrical in the GAERS model, where the occurrence of seizures was particularly prevalent at the time of the transition from activity to rest (Figure 7). Indeed, from activity to rest, the maximum amplitude of the seizing fraction increased by 4–4.5 times and remained significant for time windows in the range of 2.9–6.5 min, depending on the criteria (Figure 7A). On the other hand, the transition from rest to activity showed an opposite yet smaller effect on seizing fraction, consisting mainly in a decrease for two of the three criteria, in the range of 43–67%, over a window of 0.7–1.3 min. While transition from activity to rest occurs without seizure in a 1-min time window (null seizing fraction) in 30–45% of cases, depending on activity marker, this is about half the cases observed at distant time points from the transition (Figure 7B). This implies that seizing is not mandatory for the transition from activity to rest but is a strong, specific correlate.

Our third finding, that GAERS have prolonged periods of activity and rest compared to controls, suggests that their circuits that are supposed to trigger a cessation of activity either do not detect when rest is needed or are inefficient at triggering rest. Such pathways of central fatigue include complex multifactorial mechanisms based on monoamines and cytokines (37), which play a key role in wake/sleep switching and global metabolic homeostasis. Indeed, we expected that GAERS would show more frequent rest periods due to the freezing behavior observed during electrographic seizures. Rather, isolated seizures only transiently reduce some markers of activity. Mechanisms underlying SBG appear to be a more likely link between absence seizure and rest debts. Thus, the global picture emerging from our data suggests that GAERS have a deficiency in the mechanisms of pausing activity when fatigue accumulates. A subsequent fallback mechanism lasting a few minutes could force the brain network to rest. The corresponding delayed switch to rest is associated with an increased occurrence of seizures. This conceptual framework may help redirect research into the treatment of absence epilepsy toward rescuing resting triggers rather than blocking seizures since seizures may be more than just a symptom but rather a beneficial aid to resetting cerebral dynamics (38, 39) and metabolic status.

### Seizure clusters and seizure precursor signs

Our results show that seizures in GAERS do not occur as a time-independent (Poisson) process. Rather, our observations suggest that seizure occurrence is controlled both by a reflexive effect from the past history of seizure and by an external influence from the activity/rest cycle.

While the concept of seizure cluster has always belonged to the vocabulary of clinical epileptology, it is not associated with absence epilepsy but rather is used for *grand mal*, i.e., tonic–clonic, focal, or idiopathic generalized epilepsy, where it represents an acute clinical episode of deterioration in seizure control (7, 40). Indeed, there is a history of research in the regularities and forecasting of *grand mal* seizure, aiming at alleviating the burden of the disease, using automatic signal processing on the EEG made possible by the progress in microelectronics, at least since the 1970s (41, 42). The rationale is that seizures may build up progressively before onset through progressive synchronization of assemblies of neurons, which in some patients corresponds to anticipatory symptoms such as auras (43) and thus could be detected from the EEG, even without patient notice. A major conceptual step was introduced in the 1980s, when the problem was approached with theoretical tools developed in the frame of the physics of non-linear dynamic systems (44–47), which established both the principle of predictability horizons and provided qualitative analysis of complex signals such as the EEG. Early studies showed promising scores in terms of sensitivity and specificity, yet rigorous methodological design of evaluation suggested that more developments, in terms of algorithms and statistical approach, and/or by aggregating multimodal sensors such as body parts motion detectors or video monitoring, could help improve practical suitability (48–55). In this context, our study suggests that there is a potential for such a multimodal approach in seizure prediction, although applied to *petit mal*, i.e., absence, non-convulsive epilepsy, which is significantly different from *grand mal*, tonic–clonic epilepsy, most studied in seizure prediction.

In our model of absence epilepsy, the statistical concept of periods with an increased probability of seizing applies to the pattern of seizures on a shorter time scale of minutes rather than days or weeks. Time non-independence is readily visible in the palisade representation (33) when transposed to GAERS seizure trains. Previous physiological studies have found seizure precursor EEG or neurovascular signals a few seconds or at most tens of seconds before seizure onset (19, 34, 35, 56–59). Thus, our approach, taking into account a series of seizures and global behavior, could help find earlier seizure signs. Although absence seizures cannot be considered a point process since seizure have a non-negligible duration in tens of seconds, their duration remains short compared to periods of enhanced occurrence, which count in minutes (Figures 5, 6). Combining all these monitoring approaches could allow future advanced mathematical time-process studies to help predict seizure occurrence over a larger time window of a few minutes.

In conclusion, altered patterns of activity/rest cycle, seizure occurrence clustering, and increased seizing at transition from activity to rest are temporal patterns highlighted in this model of absence epilepsy that open new venues for predicting seizure onset in patients on a time scale allowing immediate therapeutic intervention and directing studies toward the underlying mechanisms of absence epilepsy.

## Data availability statement

The raw data supporting the conclusions of this article will be made available by the authors, without undue reservation.

## Ethics statement

The animal study was approved by Ethics committee Charles Darwin No. 5 and the French Ministry for Research. The study was conducted in accordance with the local legislation and institutional requirements.

## Author contributions

HT: Conceptualization, Formal analysis, Investigation, Methodology, Writing – original draft, Writing – review & editing, Funding acquisition. REM: Conceptualization, Formal analysis, Investigation, Methodology, Funding acquisition, Writing – original draft, Writing – review & editing. AB: Conceptualization, Formal analysis, Investigation, Methodology, Supervision, Writing – original draft, Writing – review & editing, Funding acquisition. IC: Conceptualization, Data curation, Formal analysis, Funding acquisition, Investigation, Methodology, Project administration, Software, Supervision, Writing – original draft, Writing – review & editing.
